# Effects of corn variety in whole-plant corn silage on dry matter intake, average daily gain and gastrointestinal tract bacteria and metabolites in Hu lambs

**DOI:** 10.3389/fmicb.2025.1465078

**Published:** 2025-05-30

**Authors:** Jianxin Jiao, Shumin Ma, Ting Jiao, Shangli Shi, Yongquan Gao, Xia Zhang, Shengguo Zhao, A. Allan Degen

**Affiliations:** ^1^College of Pratacultural Science, Gansu Agricultural University, Lanzhou, China; ^2^Key Laboratory for Grassland Ecosystem, Ministry of Education, Gansu Agricultural University, Lanzhou, China; ^3^Sino-US Grassland Animal Husbandry Sustainable Development Research Center, Gansu Agricultural University, Lanzhou, China; ^4^College of Animal Science and Technology, Gansu Agricultural University, Lanzhou, China; ^5^Desert Animal Adaptations and Husbandry, Wyler Department of Dryland Agriculture, Blaustein Institutes for Desert Research, Ben-Gurion University of the Negev, Beer Sheva, Israel

**Keywords:** whole-plant corn silage, corn varieties, gastrointestinal tract, fermentation parameters, microorganisms, metabolites

## Abstract

**Introduction:**

Whole-plant corn silage (WPCS) is an important roughage source in ruminant nutrition, and its nutritional value can vary significantly with corn variety. Understanding how different WPCS varieties influence gastrointestinal microbiota and metabolic profiles is essential for optimizing feed efficiency and animal health.

**Methods:**

This study examined the effects of three corn varieties (2 introduced - Tunyu 168: TY; Yu silage 23: YQZ, and 1 local Longsheng 1: LS) in WPCS on gastrointestinal bacteria and metabolites in lambs. Thirty 4-month-old female Hu lambs (19.6 ± 0.26 kg) were assigned randomly to three groups (*n* = 10 per groups). After 90 days, 6 random lambs from each group were slaughtered, and contents from the rumen, ileum and cecum were collected.

**Results:**

The LS silage had the highest crude protein (CP) content, the TY silage had the lowest neutral detergent fiber (NDF) content, and the YQZ silage had the highest ammonia nitrogen (NH_3_-N) content. Dry matter intake (DMI) was greater in lambs fed the YQZ and TY silages than the LS silage, while average daily gain (ADG) was greater in lambs fed the TY silage than the YQZ and LS silages. The greatest concentration of total volatile fatty acids (TVFAs) in the rumen was measured in lambs fed the YQZ silage, and in the ileum and cecum was measured in lambs fed the TY silage. Lambs fed the YQZ silage increased the relative abundances of bacteria that degrade carbohydrates and synthesize volatile fatty acids (VFAs) in the gastrointestinal tract, and decreased the relative abundances of pathogenic bacteria in the rumen; while lambs fed the TY silage increased the relative abundances of bacteria in the cecum that degrade carbohydrate, protein and starch, and decreased the relative abundances of pathogenic bacteria in the rumen. The pathways of nicotinate and nicotinamide metabolism and folate biosynthesis were upgraded with the TY silage; whereas, pentose phosphate metabolism, histidine metabolism and folate biosynthesis were upgraded with the YQZ silage.

**Conclusion:**

These findings suggests that the YQZ and TY silages mediate rumen fermentation by altering rumen bacterial populations and metabolic activities, thereby maintaining rumen health and improving lamb growth performance. Lambs fed the TY silage had the greatest ADG and best feed conversion ratio (FCR: DMI/ADG), but the YQZ silage may have greater potential in sheep as it mediates a wider range of metabolic pathways.

## 1 Introduction

Driven by a growing demand for animal-based proteins, advancements in breeding techniques and technology, and substantial government support, China has established itself as the world’s largest producer of livestock over the past few decades (Bai et al., 2018). Per capita, meat consumption in China increased by 390% from 1980 to 2010, the largest increase over this period worldwide (Bai et al., 2018). This was due mainly to a shift in dietary patterns that included more meat, especially an increased consumption of pork (He et al., 2016). However, it is believed that China’s livestock production efficiency still lags behind some developed countries (Thornton, 2010). Improving livestock production efficiency is particularly important in the face of the growing world population and the increase in consumption of animal products (Wilkinson and Lee, 2018; Gao et al., 2023).

Ruminants play a major role in the human diet, producing nearly all the global milk and 29% of the global meat (Gerber et al., 2013). High quality feed is the key to maintaining and improving ruminant production, and currently, corn silage is the most popular high quality feed for ruminants. Whole-plant corn silage (WPCS), which includes the stalk, leaves, ears, and kernels, is known for its relatively low cost, high yield, and rich nutrients content (Ferraretto et al., 2018; Zhao et al., 2022). The area allocated for corn production for silage in China reached 16,700 km^2^ in 2020 (Zhao et al., 2022), but still trails behind the 24,000 km^2^ in the United States (Bernard and Tao, 2020).

The processing (Johnson et al., 2002), fermentation time (Rossi et al., 2023), and the addition of microbial inoculants (Guo et al., 2022) all affect the digestibility of corn silage. Corn silage, with its high content of readily fermentable starch and its capacity to optimize nutrient synchronization in the rumen, can improve the efficiency of microbial protein synthesis (Givens and Rulquin, 2004). In sheep, WPCS increased feed intake (Gao et al., 2023), and, in beef cattle, it reduced the acetate-to-propionate ratio and improved growth performance and rumen fermentation by altering the rumen microbiota and regulating amino acid, nucleotide, and carbohydrate metabolisms (Cui et al., 2022).

Limited studies have focused on the effect of the variety of corn in WPCS on rumen fermentation (Iranmanesh et al., 2023), and on the effect of WPCS intake on microbial changes in different sites of the gastrointestinal tract in sheep (Huang et al., 2021). This is particularly important today as new corn varieties are being introduced and they should be assessed. In 2023, the China’s National Crop Variety Approval Committee approved 37 new varieties of corn in an attempt to increase the quality and yield (OFI, 2023). In addition, there is a lack of information on the impacts of WPCS from different corn varieties on the rumen health of lambs. This study addresses these gaps by examining the effects of WPCS from different corn varieties on Hu lambs. Unlike previous studies, the present study integrates microbiome and metabolomic analyses to examine the microecological mechanisms driving the responses of the lambs. We hypothesized that the variety of corn in WPCS fed to lambs affects: (1) the growth performance and feed intake; and (2) bacteria and metabolites among different sites in the gastrointestinal tract. We tested these hypotheses by offering lambs WPCS with three different varieties of corn, and determined the dry matter intake (DMI), average daily gain (ADG) and feed conversion ratio (FCR: DMI/ADG), as well as the bacterial communities and metabolites at different sites of the gastrointestinal tract.

## 2 Materials and methods

### 2.1 Experimental design and animal management

All procedures on the lambs were approved by the animal ethics committee of Gansu Agricultural University (Protocol no. GAU-LC2020-27).

Thirty, 4-month old female Hu lambs with similar body condition and bodyweight (19.6 ± 0.26 kg) were divided randomly into 3 groups (*n* = 10 per group). The lambs were penned individually (2.5 m × 1.5 m), and each lamb received WPCS with one of three corn varieties and water *ad libitum*, and 515 g dry matter (DM) concentrate per day for 90 d. The three corn varieties were Longsheng 1 (LS), a local variety produced primarily for grain, and Tunyu 168 (TY), and Yu silage 23 (YQZ), two introduced varieties. Feed was offered at 08:00 and 18:00 daily, and orts were weighed before morning feeding to determine feed intake. The lambs were weighed before morning feeding on days 1, 16, 31, 46, 61, 76 and 91 of the study to determine ADG.

TY, YQZ and LS are planted widely in Huishi Town, Huining County, and Gansu Province (Table 1). These corn varieties were harvested between the late milk ripening and early wax ripening stages, when the stubble height was approximately 10 cm and the moisture content was 65% ~ 70%. The whole corns were chopped into 2–5 cm pieces, placed into blue cylinder polyethylene silage bags, and fermented for 60 days. Nutrient contents of the three varieties of ensiled corn are presented in Table 2, and the composition and nutrients of the concentrate supplement are presented in Table 3. The concentrate supplement was formulated according to United States National Research Council (NRC) (2007) standards for meat sheep, specifically for fattening 20 kg lambs at an ADG of 200 g by providing energy for growth of 1.76 MJ/d.

**Table 1:** Corn varieties, their producers and their characteristics.

Corn variety	Producer	Characteristics
TY	Beijing Tunyu Seed Industry Co., Ltd.	TY is produced for both grain and silage. The seedling leaf sheath is deep purple, leaf blades are green, leaf margins are purple, husks of the male spike are green, anthers are deep purple, and silks of the female spike are light purple. The plant type is compact, with a height of 260 cm, and the cob is positioned at 124 cm. The ear is 18.4 cm long, with 18 rows of kernels, 37 kernels per row, and a seed setting rate of 83.6%. The grains are yellow and semi-dent shaped.
YQZ	Henan Da Jing Jiu Seed Industry Co., Ltd.	YQZ is produced mainly for silage. The seedling leaf sheath is purple, leaf blades are deep green, leaf margins are purple, anthers are yellow, and husks are purple. The plant type is semi-compact, with a plant height of 330 cm, and mature plants have 18–19 leaves. The ear is 22 cm, with 12–14 rows of kernels, 42 kernels per row, and a seed setting rate of 83.5%. The grains are yellow and dent-shaped.
LS	Jinzhong Longsheng Seed Industry Co., Ltd.	LS is produced mainly for grain. The first leaf sheath of the seedling is purple, with a rounded to spatulate tip, and green leaf margins. The plant type is semi-compact, with a total of 20 leaves, a plant height of 287 cm, and a cob height of 118 cm. The anthers are purple, husks are green, silk is pink, ears are conical, cob axis is red, ear length is 20.8 cm, there are 16 rows of kernels, with 40 kernels per row, and a seed setting rate of 86.8%. The grains are yellow and dent-shaped.

**Table 2:** Nutrient contents of whole plant corn silage with three varieties of corn (g/kg DM).

Item	Corn variety			SEM	*p*-value
	YQZ	TY	LS		
DM	327^b^	328^b^	354^a^	4.86	0.005
CP	64.8^b^	65.4^b^	69.0^a^	0.79	0.035
NDF	466^a^	436^b^	472^a^	6.68	0.030
ADF	269	258	267	3.49	0.455
Ash	39.5^b^	44.0^b^	49.5^a^	1.61	0.007
Ca	4.04	3.36	4.18	0.199	0.204
P	2.43	2.45	3.05	0.132	0.065
NH_3_-N, mg/100 mL	1.20^a^	1.00^b^	1.05^b^	0.031	0.001
pH	4.09^a^	4.05^b^	4.10^a^	0.009	0.011
Lactate, ug/mL	7.25	9.52	8.08	0.434	0.073
Acetate, mmol/L	13.7^b^	15.8^a^	10.1^c^	2.53	0.000

*n* = 3. Means with different superscript letters within the same row differ from each other (*p* < 0.05). DM, dry matter; CP, crude protein; NDF, neutral detergent fiber; ADF, acid detergent fiber; NH_3_-N, ammonia nitrogen.

**Table 3:** Composition and nutrient contents of the concentrate supplement.

Items	g/kg DM
Ingredients	
Corn	485
Wheat bran	97.1
Soybean meal	155
Flaxseed cake	155
Cottonseed meal	68.0
Premix^1^	38.8
Nutrient levels	
DM (g/kg FM)	883
CP	147
Ca	17.5
P	9.20
NDF	372
ADF	132

1 The premix provided the following per kg of diets: Cu 200–550 mg; Fe 1,600–5,000 mg; Zn 1800–4,500 mg; Se 8–20 mg; Co 8–30 mg; Mn 1,200–3,000 mg; I 15–50 mg; Vitamin A, 60,000–400,000 IU; Vitamin D, 35,000–90,000 IU; Vitamin E, ≥300 IU.

DM, dry matter; CP, crude protein; NDF, neutral detergent fiber; ADF, acid detergent fiber. Units for ingredients and nutrient levels are g/kg DM except for DM which is g/kg fresh matter.

### 2.2 Sample collections and chemical analyses

At the end of the 90 d feeding period, the lambs were fasted for 24 h, but with *ad libitum* access to water. Water was withheld for the last 2 h before slaughter to reduce variations in gut fill and to facilitate cleaning the internal organs and removing the pelt. Six random lambs from each group were slaughtered according to abattoir regulations. This number of lambs was based on the effect size index (d-value), which was calculated using estimated standard deviations of the means of measured variables from previous similar studies. A sample size of 6 per group per breed resulted in a d-value close to 0.5, which is a medium and acceptable effect size for statistical analyses (Sullivan and Feinn, 2012). The gastrointestinal tract was removed carefully, and samples from the rumen, ileum, and cecum (~75 g from each) were collected for analyses of contents and gene sequencing. The rumen contents were filtered through 4 layers of gauze, and the pH was measured immediately using a benchtop acidity meter (Model HI221, Hanna, Woonsocket, RI, United States). The filtered rumen fluid was then transferred to 15 mL cryovials and stored in liquid nitrogen for analysis of rumen fermentation parameters, and microbial and metabolomic sequencing. The contents of the ileum and cecum were collected directly into 15 mL cryovials for measuring pH and fermentation parameters and for determining microbial sequencing.

WPCS and concentrate feed were analyzed for DM (method 967.03), nitrogen (N; method 981.10), ash (method 942.05), calcium (method 968.08) and phosphorus (method 965.17) contents following AOAC (1990). Crude protein (CP) content was calculated as N × 6.25. Neutral detergent fiber (NDF) and acid detergent fiber (ADF) contents were determined using an Ankom 2,200 fiber analyzer (Ankom Technology, Macedon, NY), as described by Van Soest et al. (1991). The concentrations of ammonia nitrogen (NH_3_-N) and volatile fatty acids (VFAs) in the corn silage and the gastrointestinal tract were determined by the phenol hypochlorite method (Kozloski et al., 2006) and by gas chromatography (7890A, Agilent Technologies, Santa Clara, CA, United States) (Zhou et al., 2015), respectively, and the concentration of lactate in corn silage was determined by ion chromatography (Huang et al., 2021).

### 2.3 DNA extraction and 16S rRNA sequencing

Microbial genomic DNA from the rumen, ileum and cecum contents was extracted using the QIAamp DNA stool mini kit (QIAGEN, Inc., Venlo, Netherlands). The integrity and concentration of the extracted DNA were evaluated using agarose gel (1%, wt/vol) electrophoresis and a NanoDrop 2000 UV–vis Spectrophotometer (Thermo Fisher Scientific, Waltham, MA, United States), respectively. The amplicons of the V3–V4 regions of the 16S rRNA gene were prepared using the primers 338F (5’-ACTCCTACGGGAGGCAGCA-3′) /806R (5’-GGACTACHVGGGTWTCTAAT-3′). After purification and quantification, amplicons for all samples were pooled in equimolar concentrations and sequenced on an Illumina MiSeq platform at Shanghai Personal Biotechnology Co., Ltd. (Shanghai, China).

### 2.4 Sequence processing and statistical analyses

Raw sequences with a quality score < 20, length < 150 bp, and mononucleotide repeats > 8 bp were filtered by QIIME pipeline (v1.8.0). Paired-end reads were merged using FLASH (1.2.8). Operational taxonomic units (OTUs) were clustered at 97% sequence similarity by UCLUST, and then were classified by BLAST against the Greengenes Database (version 13.8). For alpha diversity analysis, the rarefaction and rank curves were plotted, and the Chao1 index, abundance-based coverage estimator (ACE) metric, Shannon diversity index, and Simpson index were tested in QIIME. The beta-diversity analysis used the *vegan* in R (version 4.4.1) to calculate Bray-Curtis distances and generate non-metric multidimensional scaling (NMDS). The results were visualized using *ggplot2*. The analysis of similarities (ANOSIM) was used to determine differences among the three groups of silages.

### 2.5 Extraction of rumen metabolites, and metabolite data preprocessing annotation and analysis

Rumen samples were prepared following Sun et al. (2015), with mobile phase A: 0.1% formic acid aqueous solution; mobile phase B: 0.1% formic acid acetonitrile; and an injection volume of 1 μL. The liquid chromatography–mass spectrometry (LC/MS) system for metabolomics analysis consisted of a Waters Acquity I-Class PLUS ultra-high performance liquid tandem Waters Xevo G2-XS QTOF high resolution mass spectrometer (Waters, Milford, MA, United States) and the Waters Acquity UPLC HSS T3 column (1.8 um, 2.1 × 100 mm).

The Waters Xevo G2-XS QTOF high resolution mass spectrometer collects primary and secondary data in MSe mode using acquisition software MassLynx V4.2 (Waters, Milford, MA, United States). In each cycle, dual-channel data were collected on both low and high collision energy concurrently. The low collision energy was 2 V, the high collision energy range was 10 ~ 40 V, and the scanning frequency was 0.2 s for a mass spectrum. The parameters of the ESI ion source were as follows: capillary voltage: 2000 V (positive ion mode) or −1,500 V (negative ion mode); cone voltage: 30 V; ion source temperature: 150°C; desolvent gas temperature: 500°C; backflush gas flow rate: 50 L/h; and desolventizing gas flow rate: 800 L/h.

The raw data collected using MassLynx V4.2 were processed by Progenesis QI software for peak extraction, peak alignment and other data processing operations, based on the online METLIN database and Biomark’s self-built library for identification. Concomitantly, theoretical fragments were identified and mass deviations were calculated. All data were within 100 ppm. The original peak area information was normalized with the total peak area.

The identified compounds were classified and pathway information was obtained in the Kyoto Encyclopedia of Genes and Genomes (KEGG), Human Metabolome Database (HMDB) and lipidmaps databases. The R language package *ropls* was used for orthogonal partial least squares-discriminant analysis (OPLS-DA) modeling, and 200 times permutation tests were used to verify the reliability of the model. The variable importance in projection (VIP) of the model was calculated using multiple cross-validation. The combined VIP, *t*-test, and fold change analysis (FC Analysis) identified the differential metabolites, with the criteria of VIP > 1.0, FC > 2 or FC < 0.5 and *p* < 0.05. The differential metabolites of the KEGG pathway enrichment were determined using the hypergeometric distribution test.

### 2.6 Statistical analyses

All data were tested for normality and homogeneity of variances and met the criteria. One-way ANOVA tested for differences in pH, NH_3_-N, and VFAs in different sites of the gastrointestinal tract among the three lamb groups (SPSS version 26.0; SPSS Inc., Chicago, IL, United States), with *p* < 0.05 accepted as the level of significance. The ADG of each lamb was determined by regressing bodyweight over time. The Metastats test in Mothur (version 1.30.1) was used to identify rumen bacteria that displayed different abundances among lamb groups. Significance was accepted at *p* < 0.05 and a risk of false discovery (q value) at < 0.10. The value of VIP was obtained from the OPLS-DA model, and variables with VIP > 1 and *p* < 0.05 (student’s *t*-test) were considered significant.

## 3 Results

### 3.1 Composition of silages and DMI, ADG, and FCR

The DM, CP and ash contents were greater (*p* < 0.01) in the LS silage than in the TY and YQZ silages. The NDF content and pH were lesser (*p* < 0.05) and acetate content was greater (*p* < 0.01) in the TY silage than the other two silages, whereas, the NH_3_-N content was greater (*p* < 0.01) in the YQZ silage than the other two silages (Table 2).

There was no effect (*p* > 0.05) of silage type on final body weight (BW) of the lambs. The DMI of the YQZ and TY silages were greater than the LS silage (*p* < 0.05); whereas the ADG was greater (*p* < 0.01) while the FCR was lesser (*p* < 0.05) in lambs fed the TY silage than the YQZ and LS silages (Table 4).

**Table 4:** Effect of whole-plant corn silage with different corn varieties on DMI, body weights, ADG and FCR in Hu lambs.

Item	Treatments			SEM	*p*-value
	YQZ	TY	LS		
Initial BW (kg)	19.8	19.7	19.3	0.250	0.723
Final BW (kg)	30.5	33.3	29.4	0.721	0.063
DMI, g/d^1^	1288^a^	1270^a^	1200^b^	0.046	0.002
ADG, g/d	148^b^	189^a^	140^b^	6.42	0.001
FCR	8.72^a^	6.88^b^	9.62^a^	0.456	0.023

1 DMI includes 515 g DM/d concentrate.

YQZ, Yu silage 23 + concentrate supplement; TY, Tunyu 168 + concentrate supplement; LS, Longsheng 1 + concentrate supplement; BW, body weight; DMI, dry matter intake; ADG, average daily gain; FCR, feed conversion ratio; SEM, standard error of the mean. Mean values with different superscripts within a row differ from each other (*p* < 0.05).

### 3.2 Gastrointestinal tract fermentation parameters

**Figure 1:**
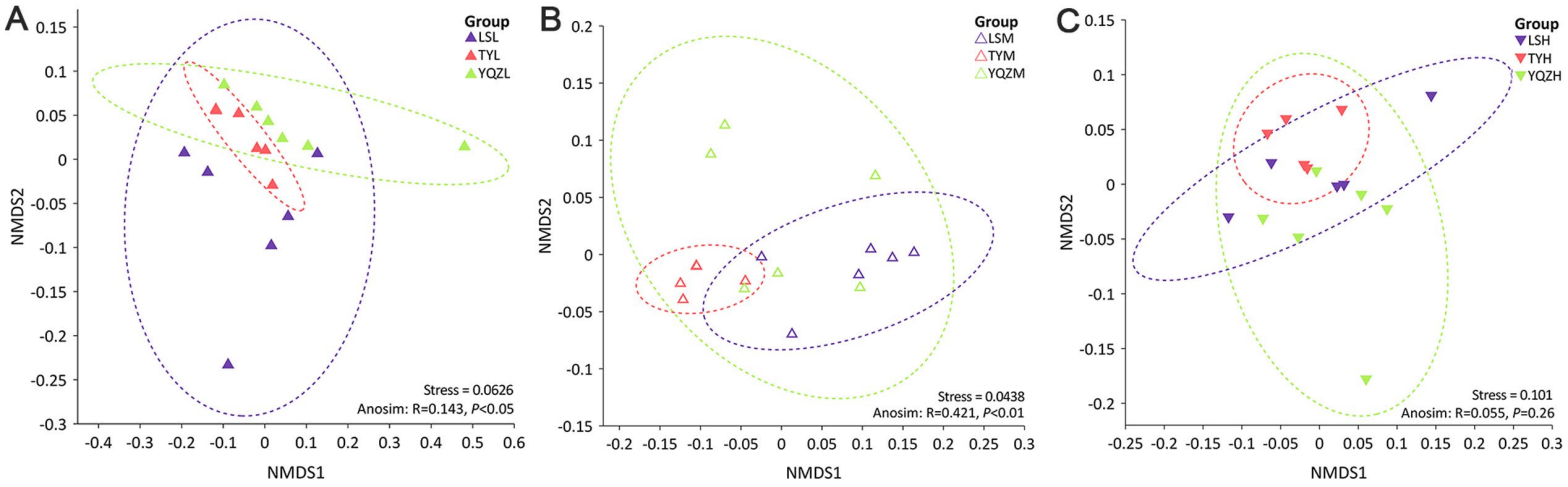
Changes in the microbial community structures of lambs in the rumen **(A)**, cecum **(B)**, and ileum **(C)** were visualized using NMDS based on Bray–Curtis dissimilarity. ANOSIM was used for statistical testing of treatment similarities. The dotted ellipse borders represent the 95% confidence interval.

In the rumen, pH was lowest and the contents of acetate, propionate, butyrate and total volatile fatty acids (TVFAs) were highest (*p* < 0.05) in lambs fed the YQZ silage, while NH_3_-N content was higher (*p* < 0.05) in lambs fed the LS than TY silage. In the ileum and cecum, pH was lowest (*p* < 0.05), but contents of acetate and TVFAs were highest (*p* < 0.05) in lambs consuming the TY silage (Table 5), whereas NH_3_-N content was highest (*p* < 0.05) in lambs consuming the YQZ silage (Figure 1).

**Table 5:** Effects of whole-plant corn silage with different corn varieties on gastrointestinal fermentation parameter in Hu lambs.

Position	Item	Corn varieties			SEM	*p*-value
		YQZ	TY	LS		
Rumen	pH	6.58^b^	6.69^a^	6.72^a^	0.026	0.038
	NH_3_-N, mg/100 mL	4.47^ab^	3.24^b^	6.31^a^	0.563	0.059
	Acetate, mmol/L	48.5^a^	35.4^b^	30.6^c^	2.68	0.000
	Propionate, mmol/L	19.6^a^	10.0^b^	10.0^b^	1.60	0.000
	Isobutyrate, mmol/L	0.62	0.76	0.65	0.033	0.216
	Butyrate, mmol/L	6.67^a^	4.45^b^	3.55^c^	0.479	0.000
	Isovalerate, mmol/L	0.78	0.88	0.75	0.028	0.101
	Valerate, mmol/L	0.54^a^	0.53^a^	0.43^b^	0.021	0.044
	A:P	2.48^b^	3.66^a^	3.06^ab^	0.021	0.028
	TVFAs, mmol/L	76.7^a^	52.1^b^	46.1^c^	4.70	0.000
Ileum	pH	7.10^b^	6.85^c^	7.22^a^	0.057	0.000
	NH_3_-N, mg/100 mL	9.84^a^	7.08^b^	5.72^c^	0.606	0.000
	Acetate, mmol/L	8.22^b^	9.57^a^	5.22^c^	0.640	0.000
	Propionate, mmol/L	ND	1.57	ND		
	Isobutyrate, mmol/L	ND	ND	ND		
	Butyrate, mmol/L	ND	0.61	ND		
	Isovalerate, mmol/L	ND	0.22	ND		
	Valerate, mmol/L	ND	ND	ND		
	A:P	ND	6.12	ND		
	TVFAs, mmol/L	8.22^b^	12.0^a^	5.22^c^	0.970	0.000
Cecum	pH	6.95^a^	6.69^b^	6.97^a^	0.048	0.002
	NH_3_-N, mg/100 mL	17.8^a^	13.2^b^	12.8^b^	0.857	0.001
	Acetate, mmol/L	57.9^b^	79.6^a^	51.0^c^	4.34	0.000
	Propionate, mmol/L	12.8^b^	16.5^a^	11.6^b^	0.776	0.001
	Isobutyrate, mmol/L	1.08^a^	0.51^b^	0.60^b^	0.084	0.000
	Butyrate, mmol/L	6.00^a^	5.36^a^	2.83^b^	0.498	0.000
	Isovalerate, mmol/L	1.21^a^	0.89^ab^	0.64^b^	0.112	0.093
	Valerate, mmol/L	0.98^a^	0.70^b^	0.90^a^	0.068	0.000
	A:P	4.52	4.82	4.41	0.086	0.118
	TVFAs, mmol/L	80.0^b^	104^a^	67.6^c^	5.37	0.000

YQZ, Yu silage 23 + concentrate supplement; TY, Tunyu 168 + concentrate supplement; LS, Longsheng 1 + concentrate supplement; TVFAs, total volatile fatty acids; A:P, the ratio of acetate to propionic acid; NH_3_-N, ammonia nitrogen; SEM, standard error of the mean. ND, not detected, the concentration of the target substance in the specific site was below the detection threshold. Mean values with different superscripts within a row differ from each other (*p* < 0.05).

### 3.3 *α*-diversity of gastrointestinal bacteria in lambs fed WPCS with different corn varieties

**Figure 2:**
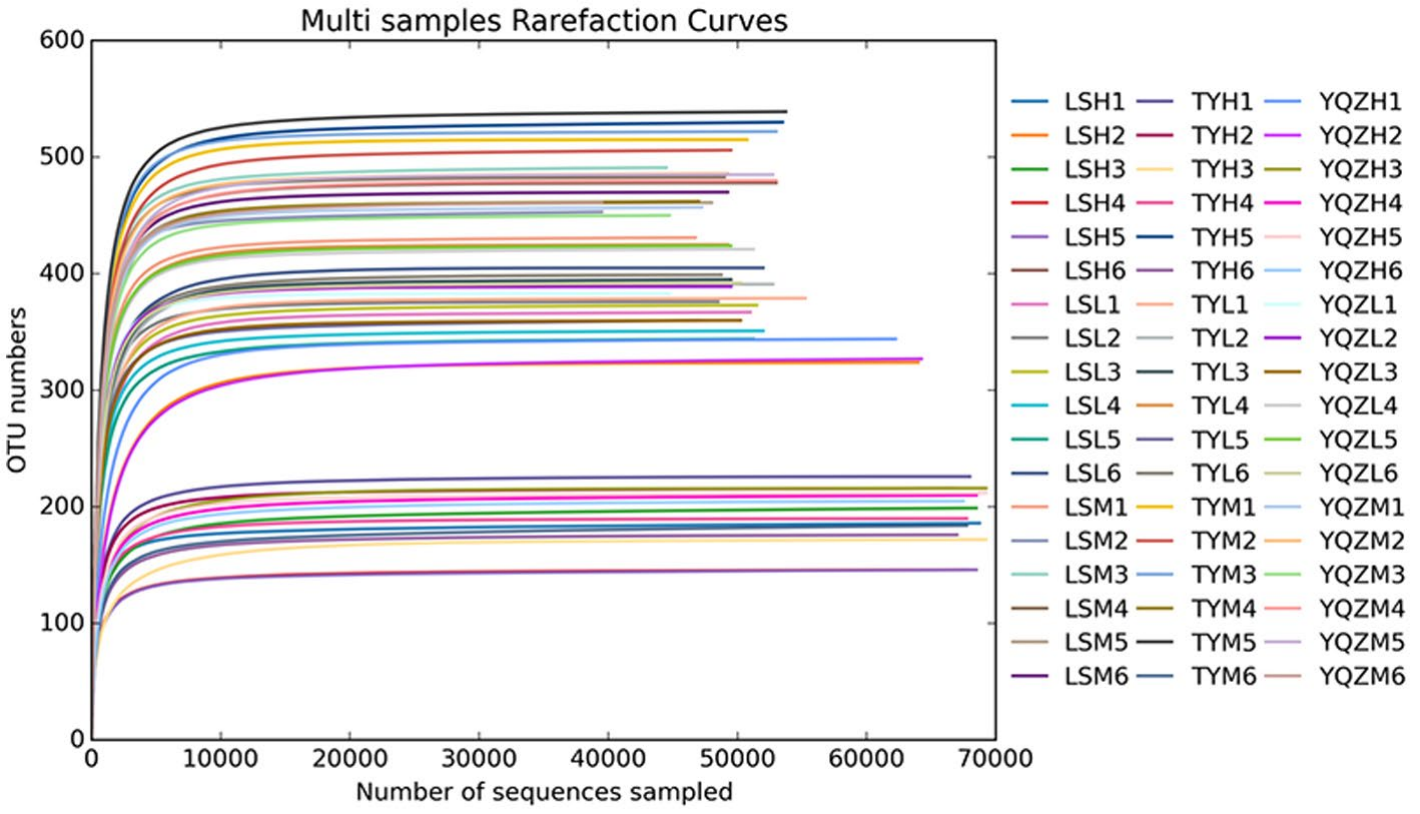
Rarefaction curves. X-axis: counts of randomly sampled sequences; Y-axis: counts of features detected by giving sequences. Lines with different colors represent different samples. YQZL (rumen bacterial diversity in YQZ), YQZH (ileum bacterial diversity in YQZ), YQZM (cecum bacterial diversity in YQZ): Yu silage 23 + concentrate; TYL (rumen bacterial diversity in TY), TYH (ileum bacterial diversity in TY), TYM (cecum bacterial diversity in TY): Tunyu 168 + concentrate; LSL (rumen bacterial diversity in LS), LSH (ileum bacterial diversity in LS), LSM (cecum bacterial diversity in LS): Longsheng 1 + concentrate.

In total, 4,303,541 raw reads were obtained for 16S rRNA bacterial genes in the three groups of lambs. After filtering, 4,286,880 effective tags were obtained, accounting for 99.6% of the raw reads. Good’s coverage values for all samples were greater than 99.9%, and, therefore, sequencing depth was suitable for analysis of the bacteria. The rarefaction curves plateaued, indicating that the sequencings were saturated and that all bacteria were identified (Figure 2). There was no difference (*p* > 0.05) among lamb groups in the α-diversity indices, including Chao1, ACE, and Simpson and Shannon indices (Table 6).

**Table 6:** Summary of alpha diversity metrics of bacteria in different sites of the gastrointestinal tract in Hu lambs fed whole-plant corn silage with different varieties of corn.

Site	Item	Corn varieties			SEM	*p*-value
		YQZ	TY	LS		
Rumen	Feature	395	392	369	5.69	0.137
	ACE	395	392	370	5.65	0.130
	Chao1	396	393	370	5.64	0.126
	Simpson	0.99	0.99	0.99	0.001	0.148
	Shannon	7.74	7.50	7.38	0.069	0.091
	Coverage	1.00	1.00	1.00	0.000	1.00
Ileum	Feature	252	252	247	25.7	0.995
	ACE	253	252	248	25.7	0.997
	Chao1	254	252	249	25.7	0.997
	Simpson	0.96	0.97	0.97	0.003	0.553
	Shannon	5.94	6.24	6.22	0.191	0.790
	Coverage	1.00	1.00	1.00	0.000	0.521
Cecum	Feature	470	455	465	17.7	0.945
	ACE	471	456	466	17.4	0.949
	Chao1	471	457	466	17.5	0.955
	Simpson	0.99	0.99	0.99	0.002	0.485
	Shannon	7.93	7.77	8.15	0.149	0.599
	Coverage	1.00	1.00	1.00	0.000	0.454

YQZ, Yu silage 23 + concentrate supplement; TY, Tunyu 168 + concentrate supplement; LS, Longsheng 1 + concentrate supplement; SEM, standard error of the mean.

### 3.4 *β*-diversity of gastrointestinal bacteria of lambs fed WPCS with different corn varieties

A NMDS compared the similarity of the gastrointestinal tract samples in terms of species diversity; the further the dispersion, the greater the differences among groups. The distances among groups were relatively close (Figure 1), and, consequently, the differences among them were small. Based on the anosim analysis, the closer the R value is to 1, the greater the difference among groups is than within groups; and a *p* < 0.05 indicates high reliability of the test. Based on the ANOSIM analysis, the differences among bacterial groups were greater than within groups in the rumen and cecum (Figure 1).

### 3.5 Effects of WPCS from different corn varieties on gastrointestinal bacteria in Hu lambs

#### 3.5.1 Effects on ruminal bacteria

**Figure 3:**
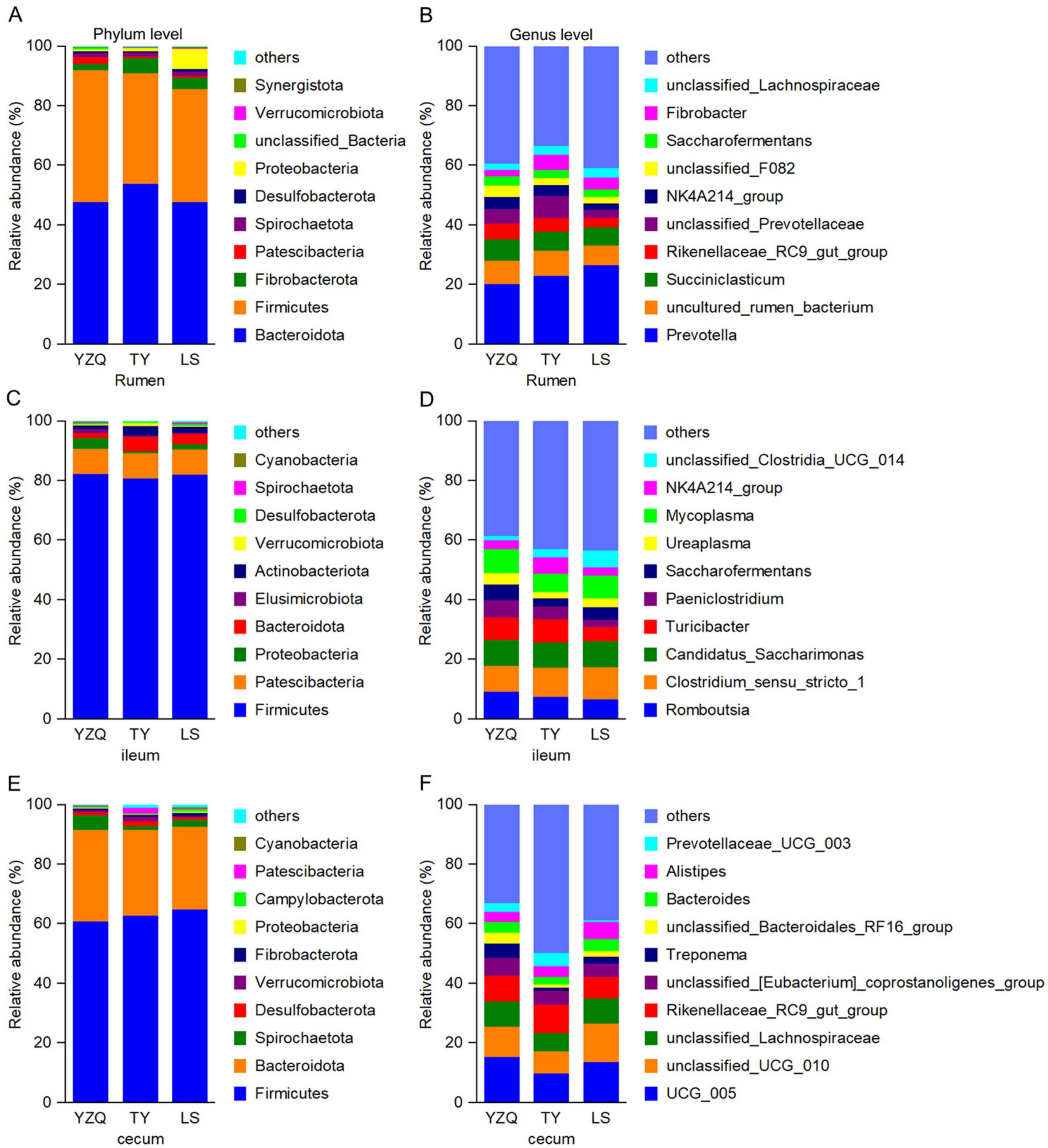
Relative abundances of bacteria at the phylum level (left) and genus level (right) in the rumen **(A,B)**, ileum **(C,D)** and cecum **(E,F)** of Hu lambs consuming whole-plant corn silage with different corn varieties.

In the rumen, Bacteroidota was the dominant phylum in the three groups, accounting for more than 47% of the total abundance, followed by Firmicutes. Proteobacteria was the third most abundant phylum with the LS silage, accounting for 6.83%, whereas, Fibrobacterota was the third most abundant phylum with the YQZ and TY silages, accounting for 2.20 and 4.95%, respectively. The relative abundance of Proteobacteria with LS silage was greater (*p* < 0.05) than with the other two silages, while Patescibacteria with YQZ silage was greater (*p* < 0.05) than with the other two silages (Figure 3A). The dominant genus in the three groups was *Prevotella*, with a relative abundance of more than 20%, followed by *uncultured_rumen_bacterium*. The third most abundant genus with the YQZ and LS silages was *Succiniclassicum*, and with the TY silage was *unclassified_Prevotella* (Figure 3B).

#### 3.5.2 Effects on ileal bacteria

In the ileum, the dominant bacterial phylum in the three groups was Firmicutes, with a relative abundance of more than 80%, followed by Patescibacteria. The third most abundant phylum with the TY and LS silages was Bacteroidota, and with the YQZ silage was Proteobacteria (Figure 3C). The dominant genus with the YQZ silage was *Romboutsia*, with a relative abundance of 11.0%, followed by *Mycoplasma*; whereas the dominant genus with the TY and LS silages was *Clostridium _sensu_stricto_1*, with a relative abundance of more than 8.0%, while the second most abundant genera with the TY and LS silages were *Romboutsia* and *Mycoplasma*, respectively. The relative abundance of *Paeniclostridium* with the YQZ silage was greater (*p* < 0.05) than with the LS silage (Figure 3D).

#### 3.5.3 Effects on cecal bacteria

In the cecum, the dominant bacterial phyla in the three groups were Firmicutes and Bacteroidota. The relative abundance of Elusimicrobiota with the YQZ silage, 0.06%, was lesser (*p* < 0.05) than with the other two silages (Figure 3E). The dominant genera with the YQZ and LS silages were *UCG_005*, *unclassified_UCG_010*, and *unclassified _Lachnospiraceae*, whereas with the TY silage were *UCG_005*, *Rikenellaceae_RC9_gut_group*, and *unclassified_ UCG_ 010*. The relative abundance of *unclassified_Bacteroides_RF16_group* with the YQZ silage was 3.62%, which was greater (*p* < 0.05) than with the other two silages; *of Prevotelaceae_UCG_003* with the TY silage was 4.42%, which was greater (*p* < 0.05) than with the other two silages; and of *Alistipes* and *Bacteroides* with the LS silage was greater (*p* < 0.05) than with the TY silage (Figure 3F).

#### 3.5.4 Bacterial species differences in the gastrointestinal tract among lambs fed different silage types

**Figure 4:**
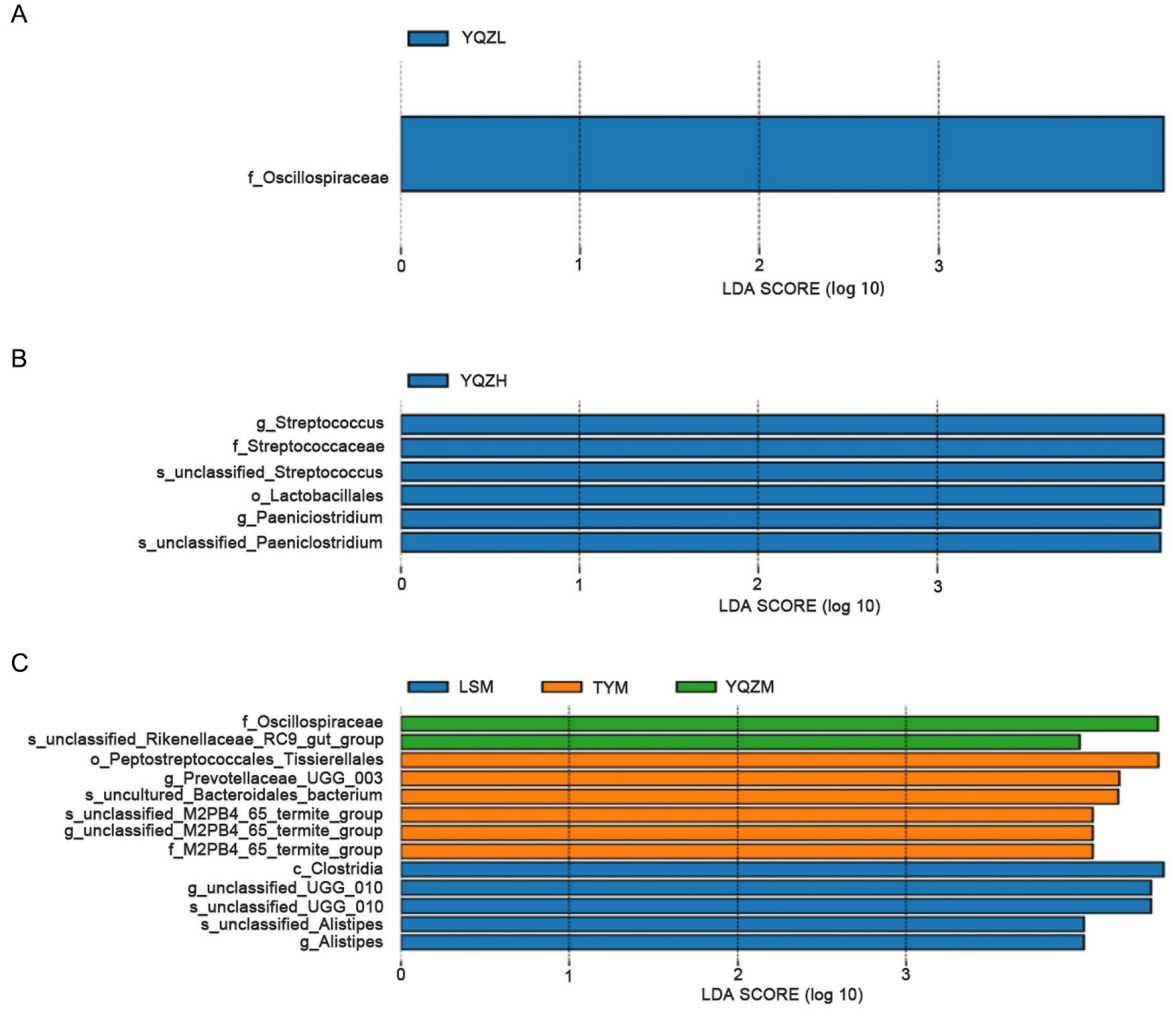
LDA value distribution histogram. The figure displays the species with LDA scores greater than the set value (the default is 4.0). The length of the histogram represents the impact of different species (i.e., LDA score), while different colors represent species in different groups. The rumen **(A)**, ileum **(B)** and cecum **(C)** are presented from top to bottom.

The LEfSe (line discriminant analysis (LDA) effect size) method was used to identify the features differing among the three lamb groups. With the default LDA score value of 4 as the standard, bacterial species with a value greater than 4 were considered to exhibit a significant effect size and were used as a biomarker. The longer the LDA histogram, the greater the impact of different bacterial species on differences among groups. The biomarker was displayed in a phylogenetic tree, and its color was determined by the color of the group in which it had the greatest influence. The LDA value distribution histogram revealed that there was one different bacteria, *Oscillospiraceae*, in the rumen, which was greater (*p* < 0.05) with the YQZ silage than with the other two silages (Figure 4). In the ileum, six differential bacteria, Streptococcaceae, *Streptococcus*, *Unclassified_streptococcaceae*, Lactobacillales, *Paeniclostridium*, and *unclassified_Paeniclostridium*, were greater (*p* < 0.05) with the YQZ silage than with the other two silages. In the cecum, there were thirteen different bacteria in the three groups. Relative abundances of *Oscillospiraceae* and *unclassified_Rikenellaceae_RC9_gut_group* with the YQZ silage were greater (*p* < 0.05) than with the YQZ and LS silages; relative abundances of *Peptostreptococcales_tissierellales*, *Prevotellaceae_UCG_003*, *uncultured_Bacteroidales_bacterium*, *unclassified_M2PB4_65_termite_group, M2PB4_65_termite_Group* were greater with the TY silage (*p* < 0.05) than with the YQZ and LS silages; while, the relative abundances of *Clostridia*, *unclassified_UCG_010*, *unclassified Alistipes* and *Alistipes* with the LS silage were greater (*p* < 0.05) than with the other two silages.

### 3.6 Effects of corn varieties in WPCS on rumen metabolites in Hu lambs

**Figure 5:**
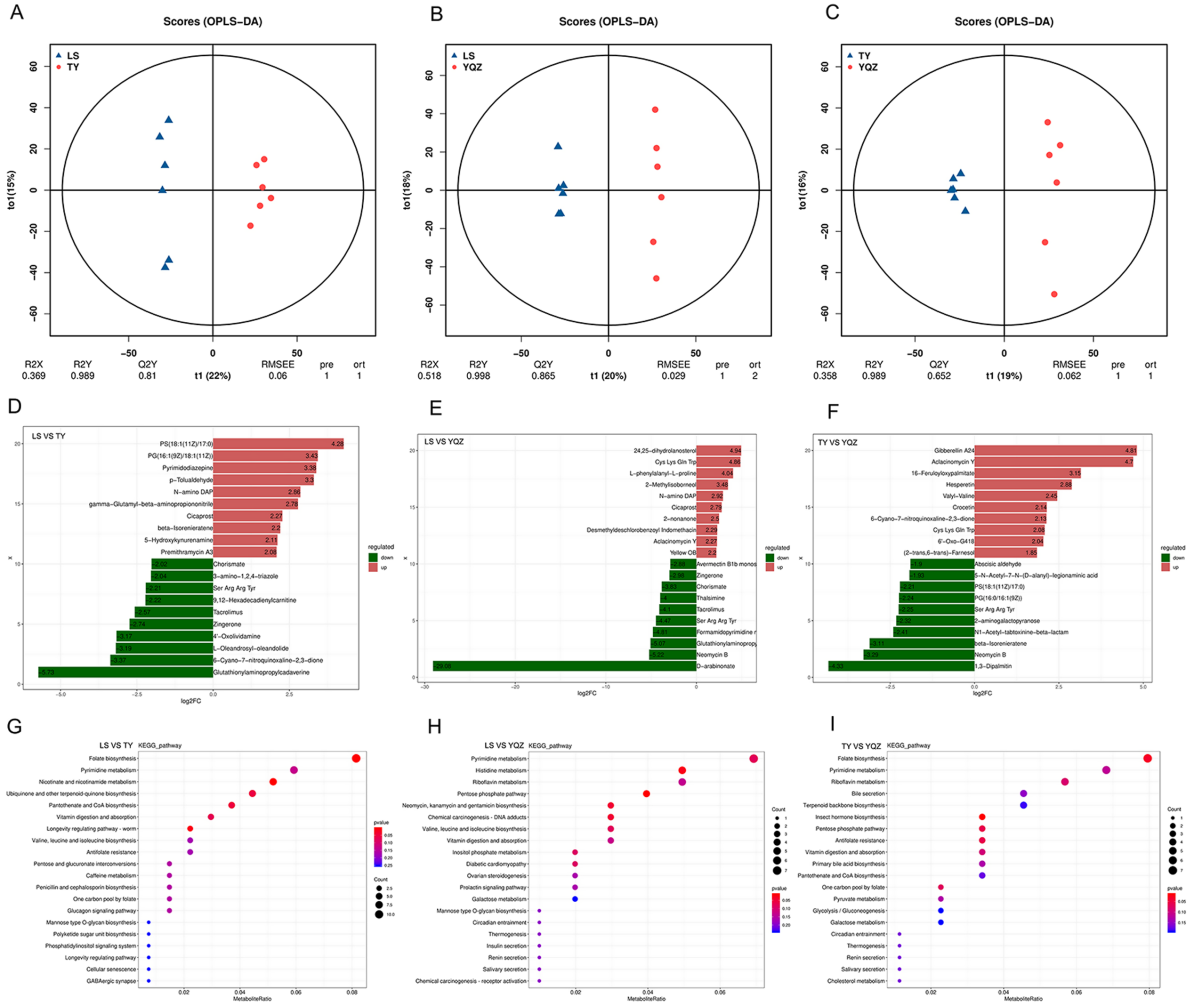
**(A–C)** OPLS-DA score diagram. The abscissa represents the score values of the predicted components; the abscissa direction indicates the difference between the groups; the ordinate represents the scores of orthogonal components; and the ordinate direction indicates the difference within the group. Percentage represents the degree of interpretation of the data set by the components; **(D–F)** Difference multiple histogram. The labels of each column represent the metabolites, which are distinguished by the up (red) and down (green) directions. The length of the column represents logFC. Only the up and down directions of 10 substances with the largest difference multiple of differential metabolites are displayed, and less than 20 substances displayed all differential metabolites; **(G–I)** Classification of different metabolite pathways in each group. The x-axis is the number of differential metabolites in the pathway, and the y-axis is the name of the pathway.

In total, 2,329 and 1793 small molecule compounds were identified under the positive and negative ion modes, and 4,122 metabolites, mainly amino acids, organic acids, fatty acids, amines, lipids, and sugars, were identified. The partial projections to late structures discriminant analysis (OPLS-DA) resulted in R^2^Y = 0.989, Q^2^Y = 0.810; R^2^Y = 0.998, Q^2^Y = 0.865; R^2^Y = 0.989, Q^2^Y = 0.652 (LS vs. TY; LS vs. YQZ; and TY vs. YQZ), indicating that (1) the interpretation and prediction of the models were strong; (2) samples of the three groups displayed significant separation; and (3) the composition of small molecular compounds in the rumen differed among the three groups (Figures 5A–C). VIP values were obtained through statistical analysis and OPLS-DA. With *p* < 0.05 and VIP > 1 as screening criteria, 989, 872 and 747 differential metabolites were identified among the three groups (LS vs. TY, LS vs. YQZ, and TY vs. YQZ, respectively) (Table 7). The metabolites with *p* < 0.05, FC > 2 or FC < 0.5 in univariate analysis of variance were selected as the final differential metabolites. Among the three groups, the top 20 metabolites were up- and down-regulated, mainly for fatty acids, organic acids, sugars, and sterols (Figures 5D–F). Compared with lambs fed the LS silage: (1) lambs fed the TY silage increased the ruminal contents of PS (18:1(11Z)/17:0), PG (16:1(9Z)/18:1(11Z)), premithramycin A3 and 5-hydroxykynenamine, but decreased the contents of glutathionyl aminopropyl cadaverine and 6-cyano-7-nitroquinoxaline-2,3-dione; and (2) lambs fed the YQZ silage increased the ruminal contents of 24, 25-dihydrolanosterol, Cys Lys Gln Trp, and L-phenylalanyl-L-proline, but decreased the contents of D-arabinone and neomycin B. In further comparisons with lambs fed the TY silage, lambs fed the YQZ silage increased the ruminal contents of gibberellin A24, aclacinomycin Y and 16-feruloyloxypalmitate, but decreased the contents of 1,3-dipalmitin, neomycin B and beta-isorenieratene.

**Table 7:** Statistical table of differential metabolites.

Group	Total	Diff	Down	Up
LS_vs_TY	4,122	989	304	685
TY_vs_YQZ	4,122	747	475	272
LS_vs_YQZ	4,122	872	266	606

Group: grouping information of differential metabolites; Total: total number of metabolites identified, Diff: number of significantly different metabolites, Down: number of down-regulated metabolites Up: number of up-regulated metabolites. YQZ, Yu silage 23 + concentrate supplement; TY, Tunyu 168 + concentrate supplement; LS, Longsheng 1 + concentrate supplement.

### 3.7 Rumen metabolic pathways in Hu lambs fed WPCS with different varieties of corn

Based on the above differential metabolites, there were three main metabolic pathways in the rumen with the LS and TY silages, namely, nicotinate and nicotinamide metabolism, and folate biosynthesis; two main metabolic pathways with the LS and YQZ silages, namely pentose phosphate pathway and histidine metabolism; and two main metabolic pathways with the TY and YQZ silages, namely insect hormone biosynthesis and folate biosynthesis (Figures 5G–I).

## 4 Discussion

The DMI and ADG of lambs fed the traditional LS silage were lesser than lambs fed the YQZ and TY silages. Charmley (2001) reported that silage intake was not affected by high concentrations of organic acids, including acetate, and by pH. Gerlach et al. (2021), however, reported that acetate levels exceeding 17 g/kg DM in silage could have a negative impact on intake. In the present study, acetate levels ranged between 10.1 and 15.8 g/kg DM, which indicates that feed intake was not affected negatively by acetate content. An increase in NDF content (Mertens, 1987) and a decrease in pH can decrease the voluntary intake of lambs. The TY silage had the lowest NDF content and pH, which could have caused the increased DMI, and, ultimately, the highest ADG. In addition, the FCR was lowest in lambs fed the TY silage, indicating that the digestibility was higher and/or the efficiency of the utilization of energy for growth of this silage was greater than for the other two silages (Kam and Degen, 1997). The lesser NDF content of the TY silage than the other two silages could be a reason for the higher digestibility of the TY silage (Miller Kokko et al., 2020). Acetate, one of the end products of ruminal fermentation and an important precursor of fat synthesis in ruminants (Cui et al., 2022), is typically associated with increased fiber-degrading bacteria in the rumen (Liang et al., 2021). In the present study, the ruminal concentration of acetate was greater with the YQZ and TY silages than the LS silage, which likely enhanced the activity of fiber-degrading bacteria, thereby improving the digestion of NDF and, subsequently, increasing DMI and ADG.

The stability of the rumen environment directly affects microbial activity and digestion. In the present study, all lamb groups maintained a rumen pH between 6.6 and 6.7, which is optimal for cellulolysis and microbial growth (Van Soest, 1994). The ruminal NH_3_-N concentrations in lambs fed the TY and YQZ silages were 32.4 mg/L and 44.7 mg/L, respectively, falling within the optimal range of 20–50 mg/L for promoting microbial protein synthesis (Schaefer et al., 1980). In contrast, ruminal NH_3_-N concentration in lambs fed the LS silage was 63.1 mg/L, slightly exceeding the optimal range, which may indicate reduced nitrogen utilization efficiency. Additionally, TVFAs are key metabolic products of carbohydrate fermentation by rumen microbes, providing an essential energy source for the host. Lambs fed the LS silage had the lowest DMI, which potentially limited microbial fermentation activity, resulting in the lowest concentration of TVFAs throughout the gastrointestinal tract (Wang et al., 2019). In comparison, the higher DMI in lambs fed the TY and YQZ silages enhanced microbial fermentation efficiency, thereby providing greater concentration of TVFAs.

The concentrations of acetate, propionate and butyrate in the rumen with the YQZ silage were greater than with the TY and LS silages, while the concentration of acetate in the ileum and cecum with the TY silage was greater than with the YQZ and LS silages. These differences were due, most likely, to the differences in organic acids of the three silages. Acetate and butyrate are the main sources of energy for ruminants, while butyrate is also involved in fatty acid synthesis. Therefore, feeding the YQZ silage could enhance the synthesis of body fat and improve production performance. The concentration of isobutyrate with the YQZ silage was greater than with the LS silage. Isobutyrate and isovalerate are branched chain VFAs, which are side products of amino acid deamination and could improve the utilization of urea N in the rumen (Liu et al., 2009).

In the present study, the dominant rumen bacteria phyla in all lamb groups were Bacteroides and Firmicutes, which was reported for Hu lambs previously (Yang et al., 2018). Bacteroides encode numerous genes involved in polysaccharide and monosaccharide metabolisms and are key degraders of complex carbohydrates (Medvecky et al., 2018). Together with Firmicutes, they ferment dietary fibers and other nutrients producing VFAs (Tremaroli and Bäckhed, 2012; Jiang et al., 2020). Proteobacteria are mainly gram-negative bacteria, and include pathogenic bacteria, such as *Escherichia coli*, *Helicobacter pylori*, and *Salmonella* spp. (Shin et al., 2015). Lipopolysaccharides (LPS) produced by Proteobacteria during cell disintegration and lysis increase the risk of metabolic diseases in ruminants (Plaizier et al., 2012). For example, elevated LPS levels exacerbated subacute ruminal acidosis and inflammatory responses in dairy cows (Rodríguez-Lecompte et al., 2014). In the present study, the relative abundance of Proteobacteria with the LS silage was greater than with the other two silages, which may have adverse effects on the lamb’s health. However, the relative abundance with the LS silage of 6.8% was considerably less than the abundance of 19%, which was reported to destabilize the microbial community (Auffret et al., 2017).

The dominant rumen genera were *Prevotella*, *uncultured_rumen_bacterium*, and *Succiniclassicum*. *Prevotella* degrades cellulose, hemicellulose, protein and starch (Li and Guan, 2017), while *Succiniclassicum* degrades cellulose or cellobiose into succinate, acetate and carbon dioxide (An et al., 2005). The relative abundance of *Oscillospiraceae*, which is important in the production of short-chain fatty acids, in particular butyrate, through the fermentation of polysaccharides (Polansky et al., 2016) was greater with the YQZ silage than with the other silages. Although the fiber content in the three silages were similar, butyrate, propionate, acetate and TVFAs were greater with the YQZ silage than with the other two silages. This would indicate a greater fiber degradation with the YQZ silage than the other silages, which was due, at least in part, to the greater relative abundance of *Oscillospiraceae*. The TVFAs not only provide close to 70% of the energy needs of ruminants and energy for gastrointestinal epithelial cells, but also inhibit the activity of pathogenic bacteria (Polansky et al., 2016). Furthermore, studies reported a positive correlation between the acetate-to-propionate ratio and *Prevotella* (Cui et al., 2022). In the present study, the acetate-to-propionate ratio and the abundance of *Prevotella* were lower with the YQZ silage than with the other two silages, indicating that the YQZ silage altered the rumen microbial community by mediating rumen fermentation.

The main difference in ileal bacteria among the three lamb groups was the greater abundances of *Streptococcus* (order Lactobacillales) and Lactobacillales with the YQZ silage than with the other two silages. *Streptococcus* degrade starch, enhancing the production of glucose, which can be absorbed by the host (Regmi et al., 2011). Lactobacillales are gram-positive probiotics colonized in the intestinal tract and produce lactate as the major end product of fermentation. The reduced pH due to the lactate inhibits the proliferation of pathogenic bacteria (Xiao et al., 2017), providing a favorable environment for intestinal growth and development for lambs consuming the YQZ silage.

In the cecum, the relative abundances of *Oscillospiraceae* and *unclassified Rikenellaceae_RC9_gut_group* were greater with the YQZ silage than with the other two silages. *Oscillospiraceae* is important in the production of VFAs, especially butyrate (Wu et al., 2021), while *unclassified Rikenellaceae_RC9_gut_group* effectively degrades soluble polysaccharides and insoluble cellulose (Zhu et al., 2021). The relative abundances of *Peptostreptococces_tissierellales*, *Prevotelaceae_UCG_003*, *uncultured_Bateroidales_bacterium*, *unclassified_M2PB4_65_termite_group*, and *M2PB4_65_termite_Group* were greater with the TY silage than the other two silages. *Peptostreptococces* metabolizes tryptophan into indole-3-acrylic acid to enhance antioxidant capacity in cells and suppress inflammation and carcinogenesis in colon epithelial cells, while *unclassified_M2PB4_65_termite_group* and *M2PB4_65_termite_Group* belong to Bacteroides, which ferment carbohydrates into VFAs (Waite and Taylor, 2015). *Unclassified_Alistipes* and *Alistipes* with the LS silage were greater than with the other two silages. *Alistipes* can lead to intra-abdominal abscesses and bloodstream infections (Parker et al., 2020), therefore the consumption of LS silage may be detrimental to the intestinal health of lambs.

Based on differential metabolite pathway analysis, compared to the LS silage, the TY silage enriched pathways related to nicotinate and nicotinamide metabolism, folate biosynthesis, and metabolism of vitamins; whereas the YQZ silage enriched pathways related to the pentose phosphate pathway and histidine metabolism. In comparison to the TY silage, the YQZ silage enriched pathways related to insect hormone biosynthesis and folate biosynthesis.

Nicotinate and nicotinamide are involved in anti-inflammatory processes and energy metabolism, and their pathways can be modulated by compounds such as saikosaponins (Ma et al., 2016). Nicotinate, a key product in this pathway, has been reported to support rumen microbial growth, contribute to microbial stability, enhance immune responses, and improve feed conversion efficiency. However, since endogenous nicotinate synthesis may not fully meet the requirements of ruminants, nicotinic acid is commonly supplemented in ruminant diets (Yang et al., 2024). Folate, likewise, plays an essential role in methylation and epigenetic regulation, thereby contributing to cellular metabolism and potentially enhancing growth performance through improved nutrient utilization (Abbasi et al., 2018). In the present study, TY silage was associated with enrichment of pathways related to vitamin metabolism, which may suggest its potential nutritional contribution to supporting growth and immune function in ruminants. These interpretations are based on metabolic pathway enrichments and literature evidence, and not by measurements in the present study measurements.

The pentose phosphate pathway, enriched in the YQZ silage group, plays a central role in glucose oxidation and energy production. Enrichment of sugar metabolism pathways may imply enhanced energy supply to the host. Additionally, the enrichment of histidine metabolism pathways in YQZ silage could have functional relevance, as histidine is known to support tissue repair and antioxidant capacity, and is considered a growth-limiting amino acid in ruminants (Holeček, 2020; Moro et al., 2020). These observations offer potential mechanistic insights, but further studies are needed to confirm their physiological significance.

## 5 Conclusion

Silage produced from the introduced TY and YQZ varieties enhanced lamb production by improving DMI, growth performance, and gastrointestinal fermentation compared to the traditional LS silage. Notably, TY silage achieved the highest ADG and best feed conversion to body weight efficiency, while the rumen microbial community with YQZ silage promoted degradation of carbohydrates and synthesis of VFAs. These findings highlight the importance of selecting appropriate corn silage varieties to optimize both production performance and animal health in lambs, offering novel insights into the interplay between dietary composition and gut microbiota.

## Data Availability

The data presented in the study are deposited in the Sequence Read Archive (SRA) repository, accession number PRJNA1215565 (SRR32121007–SRR32121060).
